# Coding-Complete Genome Sequence of a SARS-CoV-2 Variant Obtained from Raw Sewage at the University of Tennessee—Knoxville Campus

**DOI:** 10.1128/MRA.01049-21

**Published:** 2021-11-24

**Authors:** K. T. Ash, I. Alamilla, Y. Li, D. C. Joyner, D. E. Williams, P. J. McKay, B. M. Green, C. Iler, S. E. DeBlander, F. Kara-Murdoch, C. M. Swift, T. C. Hazen

**Affiliations:** a Biosciences Division, Oak Ridge National Laboratory, Oak Ridge, Tennessee, USA; b Department of Civil and Environmental Sciences, University of Tennessee, Knoxville, Tennessee, USA; c Center for Environmental Biotechnology, University of Tennessee, Knoxville, Tennessee, USA; d Department of Microbiology, University of Tennessee, Knoxville, Tennessee, USA; e Facilities Services Department, University of Tennessee, Knoxville, Tennessee, USA; f College of Natural Science, Michigan State University, East Lansing, Michigan, USA; g Battelle Memorial Institute, Columbus, Ohio, USA; h Department of Earth and Planetary Sciences, University of Tennessee, Knoxville, Tennessee, USA; i Institute for a Secure and Sustainable Environment, University of Tennessee, Knoxville, Tennessee, USA; DOE Joint Genome Institute

## Abstract

Reported here is a coding-complete genome sequence of a SARS-CoV-2 variant obtained from raw wastewater samples at the University of Tennessee—Knoxville campus. This sequence provides insight into SARS-CoV-2 variants that circulate on large college campuses but remain mostly undetected.

## ANNOUNCEMENT

Severe acute respiratory syndrome coronavirus 2 (SARS-CoV-2) (family *Coronaviridae*, genus *Betacoronavirus*) first appeared in Wuhan, China, causing the novel infectious disease coronavirus 2019 (COVID-19) (1). As the disease began to spread across the globe, many cities turned to wastewater-based epidemiology (WBE) as a method to survey their populations for the presence of the SARS-CoV-2 virus (2). Universities, and other institutions, also began to use WBE methods to closely monitor their campuses for the presence of the SARS-CoV-2 virus (3).

The wastewater surveillance of student housing at University of Tennessee (UT)—Knoxville’s campus began in September 2020. The purpose of the WBE surveillance project was to monitor on-campus student housing, in collaboration with saliva testing, to detect and isolate individuals who were infected or exposed to COVID-19. Wastewater was collected directly from 47 buildings via a dispense valve or manhole and tested once a week until June 2021.

Wastewater samples were pasteurized for 2 h at 60°C, followed by filtration through a 0.45-μM nitrocellulose filter and then a 0.22-μM nitrocellulose filter. Fifteen milliliters of the filtered samples was concentrated using the 10 kDa Amicon Ultra centrifugal filter units, and genomic RNA was extracted using a Qiagen QIAamp viral RNA minikit (4). The samples were tested for the presence of SARS-CoV-2 by reverse transcriptase quantitative PCR (RT-qPCR), which utilized TaqPath 1-step RT-qPCR master mix, CG, along with the CDC-developed primer/probe assays 2019-nCoV_N1 and 2019-nCoV_N3. Additionally, the primer/probe assay for the *Pepper mild mottle virus* (PMMoV) was used to serve as an internal positive control (4). All assays were performed at recommended conditions. Once testing was completed, the samples were stored at −80°C.

Sample UTK-840 was obtained from a sewer manhole in front of a student residential building on the UT-Knoxville campus, on 12 October 2020. This sample was selected for sequencing due to its high average quantification cycle (*C_q_*) value (*C_q_* = 24.31) from the RT-qPCR analysis. The sample was amplified using the PCR tiling ARTIC primer pools and prepared for sequencing using the NEBNext Ultra II DNA library prep kit for Illumina (NEB catalog number E7645L) (5). Sequencing was performed on a NovaSeq 6000 instrument at the Institute for Genome Sciences, University of Maryland. The genome assembly was produced using viral-ngs v1.25.0 utilizing viral-pipelines v2.1.19.0 from the Broad Institute (6). The Illumina paired-end reads were aligned to the SARS-CoV-2 reference strain (GenBank accession number NC_045512.2). The total number of reads aligned to the reference was 15,317,116, with a mean length of 249.7 bp. The mean coverage depth was 132,599.4 reads, with a standard deviation of 125,391.3 reads.

**FIG 1:**
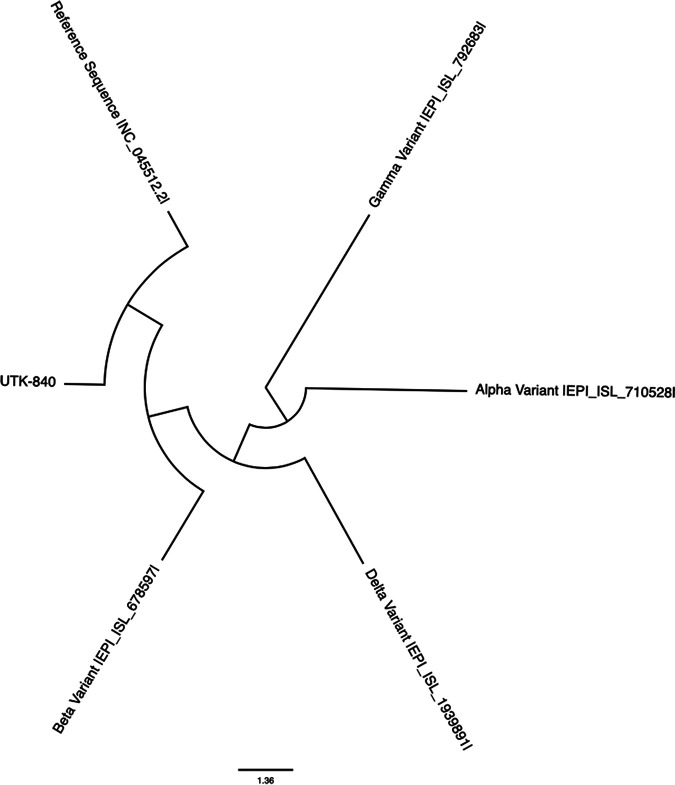
Phylogenetic tree of the UTK-840 variant in relation to the reference variant and known variants of concern. The evolutionary distance was inferred using the UPGMA method (9). The optimal tree is shown. The tree is drawn to scale, with branch lengths in the same units as those of the evolutionary distances used to infer the phylogenetic tree (10). The evolutionary distances were computed using the Jukes-Cantor method (11) and are given in the number of base substitutions per site. This analysis involved 6 whole-genomic sequences. The codon positions included were first, second, third, and noncoding. All ambiguous positions were removed for each sequence pair (pairwise deletion option). There were a total of 29,907 positions in the final data set. Evolutionary analyses were conducted using MEGA11 (12, 13).

This SARS-CoV-2 isolate is 29,874 bp long, with a GC content of 39.3%. A BLAST comparison, under default parameters, revealed that the UTK-840 sequence shares 99.93% nucleotide similarity with the Wuhan reference strain (GenBank accession number NC_045512.2) (7). The differences between the sequences are represented by 20 single nucleotide mutations, of which 15 are transition and 5 are transversion mutations. Half of these mutations occurred in the Orf1ab gene. Three mutations are in the nucleocapsid gene and only one in the spike gene, which is the D614G mutation that has been observed in most of the variants of concern. A sequence comparison made using the Audacity Instant program on GISAID, under default parameters, revealed the UTK-840 sequence to be closely related to lineage B.1.2 and the GH neighbor clade (8) (Fig. 1).

### Data availability.

The whole-genome sequence has been deposited at GenBank under the accession number MZ265375. The raw sequencing reads have been deposited at the NCBI SRA database under the accession number SRR16202037.
